# Aptamers: a novel targeted theranostic platform for pancreatic ductal adenocarcinoma

**DOI:** 10.1186/s13014-020-01624-1

**Published:** 2020-08-05

**Authors:** Q. Li, S. H. Maier, P. Li, J. Peterhansl, C. Belka, J. Mayerle, U. M. Mahajan

**Affiliations:** 1 Department of Medicine II, University Hospital, LMU Munich, Munich, Germany; 2 Department of Radiation Oncology, University Hospital, LMU Munich, Munich, Germany; 3 Department of Gastroenterology, Beijing Friendship Hospital, Capital Medical University, Beijing, China; 4 Department of Medicine A, University Medicine, Ernst-Moritz-Arndt University, Greifswald, Germany; 5 Medizinische Klinik und Poliklinik II, Klinikum der LMU München-Campus Grosshadern, Marchioninistr. 15, 81377 Munich, Germany

**Keywords:** Pancreatic adenocarcinoma, Aptamers, Theranostics, Targeted imaging, Targeted therapy, Radiation therapy

## Abstract

Pancreatic ductal adenocarcinoma (PDAC) is an extremely challenging disease with a high mortality rate and a short overall survival time. The poor prognosis can be explained by aggressive tumor growth, late diagnosis, and therapy resistance. Consistent efforts have been made focusing on early tumor detection and novel drug development. Various strategies aim at increasing target specificity or local enrichment of chemotherapeutics as well as imaging agents in tumor tissue. Aptamers have the potential to provide early detection and permit anti-cancer therapy with significantly reduced side effects. These molecules are in-vitro selected single-stranded oligonucleotides that form stable three-dimensional structures. They are capable of binding to a variety of molecular targets with high affinity and specificity. Several properties such as high binding affinity, the in vitro chemical process of selection, a variety of chemical modifications of molecular platforms for diverse function, non-immunoreactivity, modification of bioavailability, and manipulation of pharmacokinetics make aptamers attractive targets compared to conventional cell-specific ligands. To explore the potential of aptamers for early diagnosis and targeted therapy of PDAC - as single agents and in combination with radiotherapy - we summarize the generation process of aptamers and their application as biosensors, biomarker detection tools, targeted imaging tracers, and drug-delivery carriers. We are furthermore discussing the current implementation aptamers in clinical trials, their limitations and possible future utilization.

## Introduction

Pancreatic ductal adenocarcinoma (PDAC) is one of the most fatal cancers burdened with a five-year overall survival below 9% [1]. In 2018, 2.5% of the newly diagnosed cancer cases and 4.5% of all cancer-related deaths worldwide were attributed to PDAC [2]. A diagnosis at late disease stages, the lack of biomarkers for screening, early metastatic dissemination, and ultimately the resistance to systemic therapies account for the dismal prognosis of PDAC [3]. Only 20% of patients harbor resectable cancer at the time of diagnosis [4]. For 80% of patients with metastatic PDAC, the current treatment options are modified (m) FOLFIRINOX (folic acid, 5-fluorouracil, irinotecan, and oxaliplatin) or a combination of nab-paclitaxel and gemcitabine in patients with good performance status and gemcitabine with or without a second agent for those with a poor performance status [5]. Even for the fittest patients, who tolerate the most effective evidence-based treatment regimen FOLFIRINOX, the median overall survival time is only 11 months [6]. Furthermore, targeted therapies in advanced pancreatic cancer do not show significant improvement in survival [7]. Therefore, it is crucial to uncover novel and reliable biomarkers/probes for early diagnosis and surveillance. In addition, there is an urgent need to develop targeted imaging agents and drug delivery systems to improve PDAC prognosis.

Aptamers have the potential to overcome difficulties of clinical diagnosis and treatment in PDAC. These molecules are small oligonucleotide sequences that serve as ligands to target molecules such as proteins, bacteria, viruses, or cells. Due to their advantages of higher tissue penetration, rapid production, low synthesis cost, less immunogenicity, thermal stability, and ease of labeling [8], aptamers are gaining popularity as target vehicles in cancer-theranostics. Modified aptamers tagged with labeling agents function as sensitive biosensors or targeted imaging tracers. As the selection and generation can be accomplished without structural knowledge of the target molecule, aptamers can also serve as a tool to discover novel biomarkers [9]. Although they were initially conceived and designed as inhibitors, a rising number of studies reports functionally targeted agent delivery systems employing aptamers [10]. Thus, aptamers emerge as promising tools for both diagnostic and therapeutic purposes.

Since 1990, Systematic Evolution of Ligands by EXponential enrichment (SELEX) became the method of choice for generating aptamers [11]. In order to optimize that process and synthesize aptamers more reliably and efficiently, several improvements regarding binding conditions, library design, type of target, selection platform, and immobilization matrix were introduced [12]. Nowadays, with the rapid development of computer technology, the aptamer-target interactions can be predicted without affinity experiments. This allows more time- and cost-efficient selection and characterization of candidate oligonucleotides [13]. In addition to the SELEX technology, dimerization/conjugation of some aptamers increases the binding affinity and fine-tunes the target specificity, which maximizes the possibilities of various aptamer applications in the future [14].

### Aptamers as aptasensors in PDAC

Aptamers have been used as ligands for the detection step of noninvasive diagnostic assessments, such as ELISA and other immunoassays, which are usually applied for analyzing biomarkers in blood samples. These new aptamer-based assays, termed “aptasensors” can be designed to integrate readout methods, such as chemiluminescence (CL), electro-chemoluminescence (ECL), fluorescence, surface plasmon resonance (SPR), surface-enhanced Raman spectroscopy (SERS), etc. [15–17], to improve detection of existing biomarkers. Compared to traditional techniques, the unique features of aptamers, including ease of synthesis, quick turn-over time, low cost, high sensitivity, and stability under different conditions, render aptasensors a very promising alternative so that they may soon replace antibody-based assays.

Carbohydrate antigen 19–9 (CA 19–9), the only routinely used serum marker of PDAC, can specifically be bound by an aptamer, which has been identified using a trypsin-enhanced SELEX method. Although the dissociation constant (K_d_) value of 20.05 ± 3.02 nM showes that this aptasensor has a high affinity to CA 19–9, it has not been prospectively validated as aptasensor in biological fluids [18]. Carcinoembryonic antigen (CEA), another clinically established biomarker that improves the accuracy of PDAC diagnostics significantly [19], can be traced by different kinds of aptasensors [20]. Xiang and colleagues reported aptamer-based biosensors for CEA detection [21]. The performance of this aptasensor was greatly optimized and improved through the combination with nanocarriers, such as graphene, metal nanoparticles, quantum dots, etc. [20, 22]. Aptamer-based biosensors were tested for CEA measurement and showed a good selectivity, excellent stability, biocompatibility and affinity [23]. Interleukin-6 (IL-6), a major mediator of inflammation, is reported to be a diagnostic biomarker or a prognostic indicator of survival in patients with pancreatic cancer [24]. Zhuang and coworkers presented an IL-6 aptamer-based nanosensor for rapid (< 10 min), highly sensitive and specific detection of IL-6 with enhanced stability [25]. Mihaela and colleagues reported an IL-6-targeted electrochemical aptasensor based on pyrrole and gold nanoparticles, which showed high specificity and sensitivity [26]. Metalloproteinase 9 (MMP-9), another potential biomarker for diagnosis and prognostic evaluation of pancreatic cancer [27], is involved in several important processes of carcinogenesis, including invasion, metastasis and angiogenesis [28]. Scarano and coworkers developed a piezoelectric biosensor with implementation of two different aptamers in a sandwich-like approach for real-time measurement of MMP-9 [29].

Most of the other potential protein-based biomarkers for early PDAC detection, including cell migration-inducing hyaluronan binding protein (CEMIP), C4b-binding protein α-chain (C4BPA), insulin-like growth factor-binding protein 2 (IGFBP2), insulin-like growth factor-binding protein 3 (IGFBP3), interleukin-1β (IL-1β), interleukin-8 (IL-8), interleukin-10 (IL-10), vascular endothelial growth factor (VEGF), and macrophage inhibitory cytokine-1 (MIC-1) [30], could theoretically be targeted by aptamers, too. Due to a lack of large prospective clinical trials, aptamer-based biosensors directed against potential tumor biomarkers are currently only established for VEGF, a key player of angiogenesis and metastasis formation in various cancer entities [31]. VEGF aptasensors show equivalent sensing properties to VEGF antibodies and represent promising future tools of clinical diagnostics [32]. Serum tested aptasensors for the detection of established and potential PDAC biomarkers are listed in Table 1.

**Table 1:** PDAC related aptasensors for biomarkers detection

Aptamers against biomarker	Nanocarriers	Detection method	Oligos	Linear dynamic range (LDR)	Limit of detection (LOD)	Detection model	Ref.
CA 19–9	None	Fluorescence	DNA	Kd value 20.05 ± 3.02 nmol/L	–	Not tested in serum	[18]
CEA	Zirconium metal-organic framework of silver nanoclusters (AgNCs)	ECL and SPR	DNA	1.0–250 ng/mL	0.3 ng/ml	Human serum	[90]
CEA	Ru@SiO2 − AuNPs	ECL	DNA	5.0–50,000 fg/mL	1.52 fg/ml	Human serum	[91]
CEA	CdS-GR-AuNPs	ECL	DNA	0.01–10.0 ng/ml	3.8 pg/ml	Human serum	[92]
IL-6	Carbon nanotube	ECL	RNA	1 pg/mL to 10 ng/mL	1 pg/ml	Human serum	[33]
MMP-9	None	SPR	DNA	–	0.56 ng/ml	Commercial serum	[29]
VEGF	Quantum dots	Fluorescence	DNA	–	50 pmol/L	Human serum	[34]
VEGF	None	ECL	DNA	50 pmol/L to 0.15 nmol/L	5 pmol/L (190 pg/mL)	50% serum	[35]
VEGF	Carbon–gold nanocomposite	ECL	DNA	10 to 300 pg/ml	1 pg/ml	Human serum	[48]

### Aptamers for the detection of circulating cancer cells (CTCs) and novel PDAC biomarkers

The identification of promising predictive PDAC biomarkers remains challenging. Circulating tumor cells (CTCs) are single or clustered cells that loose connection to the tumor bulk and can be detected in the bloodstream. They are important biomarkers for the diagnosis and prognosis of early and metastatic cancer [36]. Thus, sensitive techniques for CTC detection would be crucial for early diagnosis, prognosis prediction, and for monitoring treatment response via noninvasive liquid biopsies. Utilizing blind cell SELEX methods, aptamers targeting cancer cell surface structures can be enriched efficiently even without knowledge of the protein expression profile of CTCs. After several rounds of positive and negative selection, aptamers are amplified, sequenced and subjected to protein mass spectrometry to identify the detailed target structure [9]. On one hand this strategy promotes the generation of specific aptamers for detection and therapeutic targeting of cancer cells, on the other hand it facilitates the discovery of novel CTC-based biomarkers.

To date, several aptamer-based biosensors have been designed to identify and quantify various CTCs with detection limits as low as one single cell [37]. In PDAC, Dua and colleagues established an RNA aptamer (SQ-2) that recognizes pancreatic cancer cells with very high specificity. In the SELEX process they used Panc-1 and Capan-1 cells for positive selection and normal human pancreatic ductal epithelial (HPDE) cells for negative selection. After sequencing the winning aptamer, they identified alkaline phosphatase placental-like 2 (ALPPL-2), an oncofetal protein, as the target of SQ-2 [38]. Mechanistic exploration of ALPPL-2 revealed its involvement in pancreatic cancer cell growth and invasion. Based on the expression of ALPPL-2 on the cell surface and as a soluble factor in the circulation, the authors also developed a sandwich-aptamer-linked immobilized sorbent assay (ALISAs) targeting ALPPL-2-positive extracellular vehicles (EVs) with high sensitivity and specificity. In conclusion, SQ-2 is a possible targeted probe for serum- and cell-based diagnostics, and ALPPL-2 appears to be a promising future biomarker for PDAC [39].

Wu and co-workers used SELEX to generate a DNA-aptamer called XQ-2d and successfully identified CD71 (transferring receptor 1) as the molecular target for cell-specific aptamer binding [40]. Other groups developed novel PDAC-cell-targeted aptamers, such as PL8, aptamer 1, and aptamer 146 [41, 42]. Even though the exact targets of those aptamers have not been identified yet, these molecules have the potential to detect CTCs. Remarkably, Kim and colleagues performed SELEX while targeting stemness-enriched PDAC cancer cells. As a result, the novel aptamer 1 and aptamer 146 were generated, which might be future candidates for the detection of cancer stem cells (CSCs) [43].

Apart from intact PDAC cells the secretomes of PDAC cells as well as PDAC tissues, were used for the SELEX process. White et al. described an in-vitro positive/negative selection strategy to identify a cyclophilin B RNA-aptamer (M9–5) that detects structural differences between the secretome of pancreatic cancer and non-cancerous cells. M9–5 has the ability to discriminate sera from PDAC-patients and healthy volunteers with high specificity and sensitivity [44, 45]. Another aptamer (BC-15), which was selected against human PDAC tissue, but not adjacent normal tissue showed a high affinity for CTCs isolated from pancreatic cancer patients. Compared to the well-established anti-cytokeratin antibody-based method, the BC-15 aptamer-based method showed similar efficacy for the identification of CTCs. Interestingly, the BC-15 aptamer exemplifies the potential to generate a particular patient-specific aptamer using individual tumor tissue as target [46]. Future applications of these personalized aptamers include precise molecular targeting or surveillance of therapeutic response.

### Aptamer-based targeted imaging of PDAC

Aptamer-based targeted imaging is one of the most promising molecular imaging technologies for PDAC diagnosis, accurate staging, and the monitoring of treatment response. Compared to conventional imaging protocols, aptamer-based targeted imaging employs tumor-specific, labeled aptamers to explore biological targets in living subjects. This method harbors the following advantages: i) early detection is much easier to achieve due to amplified imaging via aptamers targeting PDAC markers; ii) aptamers that specifically target PDAC biomarkers have the potential to differentiate between malignant and benign diseases; iii) more accurate imaging helps to evaluate precise staging of PDAC and improves surgical guidance for complete tumor resection and lymph node clearance; iv) aptamers support the detection of biological characteristics of PDAC patients for clinical decision making and prognostic prediction, which can be evaluated without biopsies [47].

**Fig. 1:**
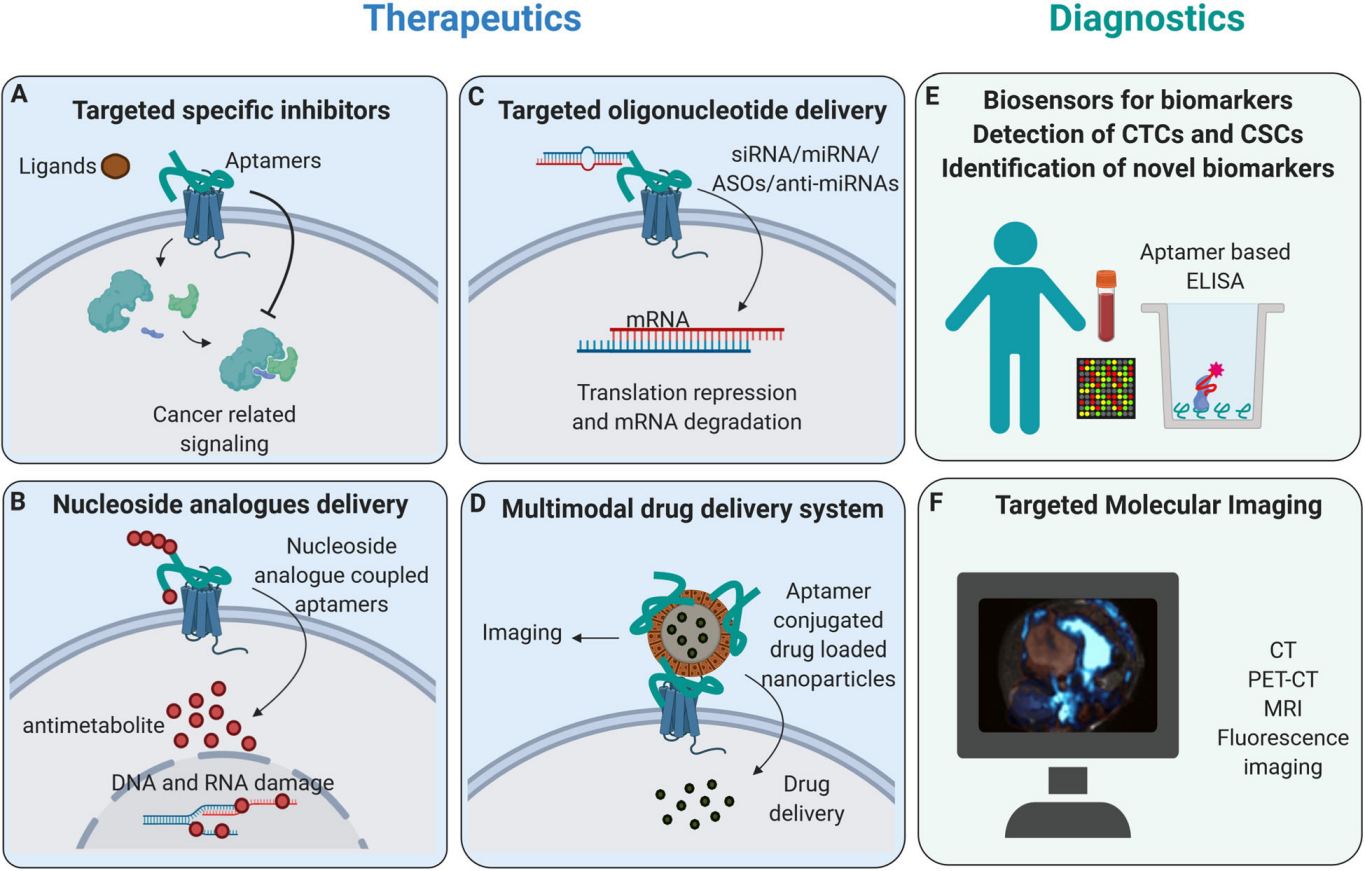
Application areas of aptamers in PDAC. Aptamers, can specifically bind target molecules and owing to their combinatorial properties for the incorporation of therapeutics and diagnostics agents, can be used as targeted theranostic (therapeutics plus diagnostics) in PDAC. **a** By specifically binding to the extracellular domain of the receptor, aptamers can work as competitive inhibitors and block cancer-related signaling in target cells. **b** Instead of nucleosides, nucleoside analogs, including gemcitabine and 5-FU, can be incorporated into aptamers. These nucleoside analog coupled aptamers can be specifically internalized and act as anti-proliferative agents. **c** Different kinds of oligonucleotides, including anti-miRNAs, ASOs, miRNAs, and siRNAs, can be incorporated into aptamers. These oligonucleotides coupled aptamers can function in targeted gene therapy in PDAC. **d** Aptamers can be incorporated into several multimodal drug delivery systems as carriers for targeted therapy and targeted imaging. **e** By integrating different readout methods, aptamers can be used as biosensors to detect defined biomarkers, CTCs and CSCs using aptamer-based ELISA methods. **f** Aptamers can be incorporated with different contrast agents to increase the sensitivity and precision of cancer detection by targeted molecular imaging. (Abbreviations: Anti-miRNAs: microRNAs inhibitors; ASOs, antisense oligonucleotides; miRNA: microRNAs; siRNA: short interfering RNAs; CTCs: cancer stem cells; CSCs: circulating tumor cells; ELISA: Enzyme-linked immunosorbent assay)

A few aptamer-based, PDAC-targeted imaging approaches have been evaluated in-vitro and in-vivo (Table 2, Fig. 1). Wang and co-workers generated a DNA-aptamer (Ap52) against the shared tumor-specific MAGE-A3_111–125_ peptide antigen. Signals of the Cy3-conjugated aptamer were specifically localized on the surface of cancer cells from seven different entities, including pancreatic cancer. These in-vitro results suggest that Ap52 may have potential for future molecular imaging [49]. In a xenograft mouse model the fluorescein-labeled aptamer XQ-2d targeting CD71 and the aptamer AP1153 targeting G-protein-coupled cholecystokinin B receptor (CCKBR) accumulated particularly in PDAC tumors [40]. Therefore, these Cy-3-labeled aptamers are promising candidates for PDAC diagnostics.

**Table 2:** PDAC related diagnostic aptamers

Name	Target	SELEX method	Positive selection	Negative selection	Oligos	Applications	Model	Ref.
M9–5	Cyclophilin B	Secretome	MiaPaCa-2	HPDE	RNA	biomarker detection	Patients and	[45]
							KPC mice serum	[44]
C14B	AGR2	Protein	AGR2-GST	GST	DNA	probe generation	In vitro	[68]
SQ-2	ALPPL-2	Cell	Panc-1	HPDE	RNA	biomarker detection	In vitro	[38]
			Capan-1			biosensor generation		[39]
BC-15	hnRNP A1	Tissue	PDAC	Adjacent normal tissue	DNA	CTCs detection	Patients serum	[46]
XQ-2d	CD71	Cell	PL45	hTERT-HPNE	DNA	biomarker detection	In vitro	[40]
						Cy-5-labeled fluorescence imaging	Xenograft model Human sections	[93]
PL8	HPAC	Cell	PL45	TOV-21G	DNA	New biomarker detection	In vitro	[41]
Apt.1	CSCs	Cell	HPAC (CRL2119)	HPDE	DNA	CSCs detection;	In vitro	[42]
Apt.146	CSCs	Cell	HPAC (CRL2119)	HPDE	DNA	New biomarker detection	In vitro	[42]
Ap52	MAGE-A3	Peptide	MAGE-A3_111–125_	None	DNA	Cy-3-labeled fluorescence imaging	In vitro	[49]
P19/P1	HPAC	Cell	PANC-1	Huh7	RNA	Cy-3-labeled fluorescence imaging	Human tumor tissue sections	[55]
AP1153	CCKBR	Peptides	CCKBR peptides	COS-1	DNA	ICG labeled fluorescence imaging	Orthotopic model	[94]
		Cell	PANC-1					
M17	MMP14	Cell	293 T-MMP14 cells	293 T cells	DNA	Cy-3-labeled fluorescence imaging	Xenograft model	[51]

Although there are no fluorophore-labeled aptamers available for clinical applications, human tumor tissue sections have been used to determine the performance of aptamer-based imaging in PDAC patient samples. Targeting PDAC cells with Cy-3-coupled P19/P1 aptamers was employed as a diagnostic tool on archival human pancreatic duodenectomy tissue sections. Scoring patterns from 72 patients revealed a positive correlation between high fluorescence signal intensity and significantly increased mortality [55]. Thus, aptamer-based, PDAC-targeted imaging seems to allow prognostic prediction.

Besides fluorescence imaging, magnetic resonance imaging (MRI) is another option for PDAC-targeted molecular imaging utilizing metal-oxide-labeled aptamers. Recently, a novel matrix metalloproteinase 14 (MMP14)-targeted aptamer (M17) conjugated with polyethylene glycol-Fe_3_O_4_ was synthesized. This molecule can specifically bind to pancreatic cancer cells in vitro and hereby reduce MRI T2-weighted imaging signal intensity [51]. As a result, this novel molecular-targeted MRI approach bears potential for PDAC diagnostics.

### Aptamer-based, molecular-targeted therapy of PDAC

In the past two decades several antibody-based, molecular-targeted therapies were implemented for the treatment of different hematologic and solid malignancies. Unfortunately, no antibody-based targeted therapy has yet been successful in improving the prognosis of PDAC patients [7]. Similar to antibodies, aptamers recognize and bind targets of interest, but they provide numerous advantages. Aptamers are small and they can be easily modified and linked to diverse nanoparticle systems for multi-functionalization. Other intrinsic features include their short production time, fully chemical synthesis, lower costs of manufacturing, no batch-to-batch variability, and better thermal stability.

Several aptamers were identified for possible treatment strategies in PDAC (Table 3). Kim and colleagues generated a 2′-fluoropyrimidine-modified RNA-aptamer (P12FR2) directed against pancreatic adenocarcinoma up-regulated factor (PAUF), a novel secretory protein overexpressed in pancreatic cancer. P12FR2 aptamers inhibited PAUF-induced migration of PANC-1 cells in vitro and decreased tumor growth by 60% in a PDAC xenograft mouse model without causing relevant weight loss in treated mice [50]. Another aptamer (P15) selected by blind PDAC cell SELEX showed significant inhibition of metastasis formation in an in-vitro assay. P15 achieves this effect by targeting the intermediate filament vimentin, an intracellular epithelial-mesenchymal transition (EMT) tumor cell marker, which is highly expressed as a mislocalized protein on the surface of pancreatic cancer cells [53]. Moreover, chemically modified aptamers against two immune checkpoint proteins, Programmed Death 1 (PD-1) and Programmed Death Ligand 1 (PD-L1) have been generated. These PD-1- and PD-L1-aptamers could mimic antibody functions in different in-vitro assays [52]. Hence, aptamers have the potential to function as antagonists or inhibitors of crucial oncogenic pathways due to their convincing therapeutic performance in preclinical PDAC models.

**Table 3:** PDAC related therapeutic aptamers and aptamers-based drug delivery systems

Targeted therapy	Target	SELEX	Preclinical model	Name	Chemistry	Ref.
Inhibitor	PAUF	Protein	Xenograft model	P12FR2	RNA	[50]
Gemcitabine	EGFR	Protein	In vitro	E07	RNA	[56]
AS1411 on gold nanoparticles (AuNS) as inhibitors	Nucleolin	Designed Aptamer	In vitro	AS1411	DNA	[61]
Doxorubicin; nanoparticles with Folic acid/AS1411	Nucleolin	Designed Aptamer	In vitro	AS1411	DNA	[64]
Doxorubicin	CD71	Protein	In vitro	C2-min	RNA	[58]
5-Fluoro-2′-Deoxyuridine	ALPPL-2	Cell	In vitro	SQ-2	RNA	[65]
Triptolide	Nucleolin	Designed Aptamer	Xenograft model	AS1411	DNA	[69]
C/EBPα -saRNA	HPAC	Cell	Xenograft model	P19/P1	RNA	[55]
C/EBPα -saRNA	CD71	Protein	Mouse model of advanced PDAC	TR14	RNA	[54]
Gemcitabine or 5-fluorouracil (5-FU)	HPAC	Cell	In vitro	P19	RNA	[66]
Inhibitor	Vimentin	Cell	In vitro	P15	RNA	[53]
Inhibitor	PD1	Protein	In vitro	XA-PD1–78	DNA	[52]
	PD-L1			XA-PDL1–82	DNA	
Monomethyl auristatin E (MMAE)	CD71	Protein	In vitro	Waz	RNA	[70]
	EGFR1			E07		
Camptothecin	Tenascin-C	Protein	Xenograft mice	GBI-10	DNA	[95]
Gemcitabine	Nucleolin	Designed Aptamer	Xenograft mice	AS1411	DNA	[63]
Doxorubicin	CD71	Cell	In vitro	XQ-2d	DNA	[93]

Oligonucleotides, such as microRNAs (miRNAs), miRNA inhibitors (anti-miRs), antisense oligonucleotides (ASOs), and short interfering RNAs (siRNAs), have been shown to potently silence the expression of their target genes. However, one of the most significant obstacles for oligonucleotide-based therapeutic strategies is the lack of specific delivery to the tumor [10]. Aptamers can recognize distinct molecules or structures on cancer cells and get endocytosed quickly upon binding. These intrinsic properties of aptamers provide a chemically modifiable option of targeted oligonucleotide delivery in the form of novel aptamer-oligonucleotide conjugates. In order to explore the performance of aptamer-oligonucleotide conjugates in PDAC therapy, small activating RNA against CCAAT/enhancer-binding protein-a (C/EBPa-saRNA) was conjugated to PDAC cell-targeted aptamers. These novel agents inhibited cell proliferation in vitro and significantly reduced tumor growth in an advanced PDAC mouse model [54, 55]. Thus, aptamer-based targeted delivery of oligonucleotides has potential therapeutic effects in advanced PDAC.

Nucleoside analogs are a class of drugs that are of special interest for aptamer-based targeted treatment. Due to the similar structure of nucleoside analogs and natural nucleosides, they can be incorporated into aptamers rather easily. Partha Ray et al. utilized a nuclease resistant RNA-aptamer that binds EGFR on pancreatic cancer cells and is subsequently internalized in order to deliver gemcitabine-containing polymers into EGFR-expressing cells, which inhibited cell proliferation in vitro [56]. Dua et al. produced an alkaline phosphatase placental-like 2 (ALPPL2) targeted RNA aptamer (SQ2) and coupled five repeats of 5-fluoro-2′-deoxyuridine (5FdU) to the 3′-end. Hence, one aptamer can deliver five monomer units of the drug, while the phosphorothioate backbone ensures that the drug is not cleaved extracellularly by the action of serum nucleases [65]. Yoon and co-workers developed a PDAC targeted RNA-aptamer (P19), which was enriched with gemcitabine or 5-fluorouracil (5-FU). They used gemcitabine triphosphate (dFdCTP) or 5-fluorouracil (5-FU) triphosphate (5FdUTP) to replace cytidine triphosphate (CTP) or uridine triphosphate (UTP) during RNA-aptamer synthesis. These aptamer-drug conjugates (ApDCs) did not only significantly inhibit cell proliferation in PANC-1 cells, but also inhibited cell proliferation in the gemcitabine-resistant pancreatic cancer cell line AsPC-1 [66]. Coincidentally, Park and colleagues created an aptamer called APTA-12 by single substitution of a guanine residue with a gemcitabine phosphoramidite at position 14 of the AS1411. APTA-12 notably inhibited the growth of pancreatic cancer models in vitro and in vivo [63]. Thus, the gemcitabine- or 5-FU-incorporated aptamers represent attractive tools for cancer cell-specific chemotherapeutic drug delivery in PDAC.

The original AS1411 aptamer mentioned above is a 26-nucleotide guanosine-rich DNA-aptamer with high affinity and specificity to nucleolin, a cell-surface receptor overexpressed in cancer cells. Because AS1411 has not only proven anti-cancer effects in models of various cancer entities, but is also an enhancer of cellular uptake, it is frequently used for the generation of aptamer-based drug delivery systems [57]. According to expectations, it was reported that an increased loading density of AS1411 on gold nanostars (AuNS) rises the quantity of AS1411 delivered into pancreatic cancer cells, which finally results in substantial pancreatic cancer cell death [61]. Lale et al. developed a dual-targeted, pH-sensitive, biocompatible polymeric nano-system conjugated with doxorubicin. This dual approach with folate and the AS1411 aptamer successfully increased the cancer-targeting efficiency of the nanoparticles resulting in a higher payload of doxorubicin in PDAC cell lines [64]. Also, the herb-derived compound triptolide (TP) can also increase anti-tumor activity in vitro and in vivo when coupled to an AS1411-linked polymeric nanocarrier [69]. These studies open new perspectives for overcoming drug resistance of pancreatic cancer.

CD71 (transferrin receptor) and EGFR are also commonly used targets in aptamer-guided therapy of PDAC. Several groups loaded doxorubicin, monomethyl auristatin E (MMAE), or monomethyl auristatin F (MMAF) to CD71- or EGFR-targeted aptamers, respectively [58, 70]. Their ability to dampen the proliferation of pancreatic cancer cells makes these aptamers attractive options for delivery of toxic substances specifically into PDAC cells.

### Aptamers in clinical trials

Since pegaptanib, an RNA-aptamer directed against VEGF_165_, was approved by the US Food and Drug Administration (FDA) as an anti-angiogenic treatment for neovascular (wet), age-related, macular degeneration (AMD) in 2004, an increasing number of aptamers have successfully entered clinical trials. Until now, three aptamers have entered phase III. The protagonists are Pegnivacogin (RB006), a direct factor IXa inhibitor; E10030, an anti-platelet-derived growth factor (anti-PDGF-B) aptamer; and Zimura, a complement factor C5-inhibitor [59].

So far, NOX-A12 is the only aptamer undergoing a clinical trial for pancreatic cancer [8]. NOX-A12 is an RNA-aptamer that targets CXCL12 (C-X-C Chemokine Ligand 12), a key chemokine protein involved in tumor cell proliferation, the formation of new blood vessels and metastasis. As a novel CXCL12 inhibitor, NOX-A12 was found to be safe and well-tolerated by 28 patients with relapsed or refractory multiple myeloma in a phase 2a study (NCT01521533) [62]. Twenty patients with metastatic microsatellite-stable (MSS) colorectal or pancreatic cancer were recruited in the Opera study (NCT03168139) to evaluate the efficacy of NOX-A12 plus pembrolizumab (PD-1 antibody) treatment. The results demonstrated that 25% of patients achieved stable disease and 35% of patients showed a prolonged survial time on treatment in comparison to the prior line of therapy. The safety profile of the combination therapy was consistent with that of pembrolizumab alone in advanced cancer patients. Therefore, NOX-A12 displayed both safety and therapeutic potential in combination with pembrolizumab. More clinical trials are needed to explore the efficacy of several more aptamers in PDAC treatment [67].

### Aptamers in combination with radiation therapy

In order to improve the therapeutic outcome of clinical PDAC treatment, it seems inevitable to design novel strategies that combine different treatment modalities aiming at achieving synergism [60]. However, the improved efficacy of combined systemic therapies often comes at the costs of severe side effects. Accordingly, local therapies move into the focus of combined modality settings. Radiotherapy is a longstanding and essential component of multimodal cancer treatment [60]. For many solid tumor entities, combination regimens of chemo- and radiotherapy represent the standard of care (e.g., locally advanced head and neck cancers or glioblastoma) [71]. However, for the treatment of PDAC, radiotherapy is rather infrequently applied, because the majority of patients present in disseminated disease stages, and PDAC is well known for its high degree of radioresistance [72]. Aptamers represent versatile tools to bypass this radioresistance due to their high target specificity, straightforward synthesis, stability in different conditions and body fluids and the abundant spectrum of possible modifications as described in the previous sections.

**Fig. 2:**
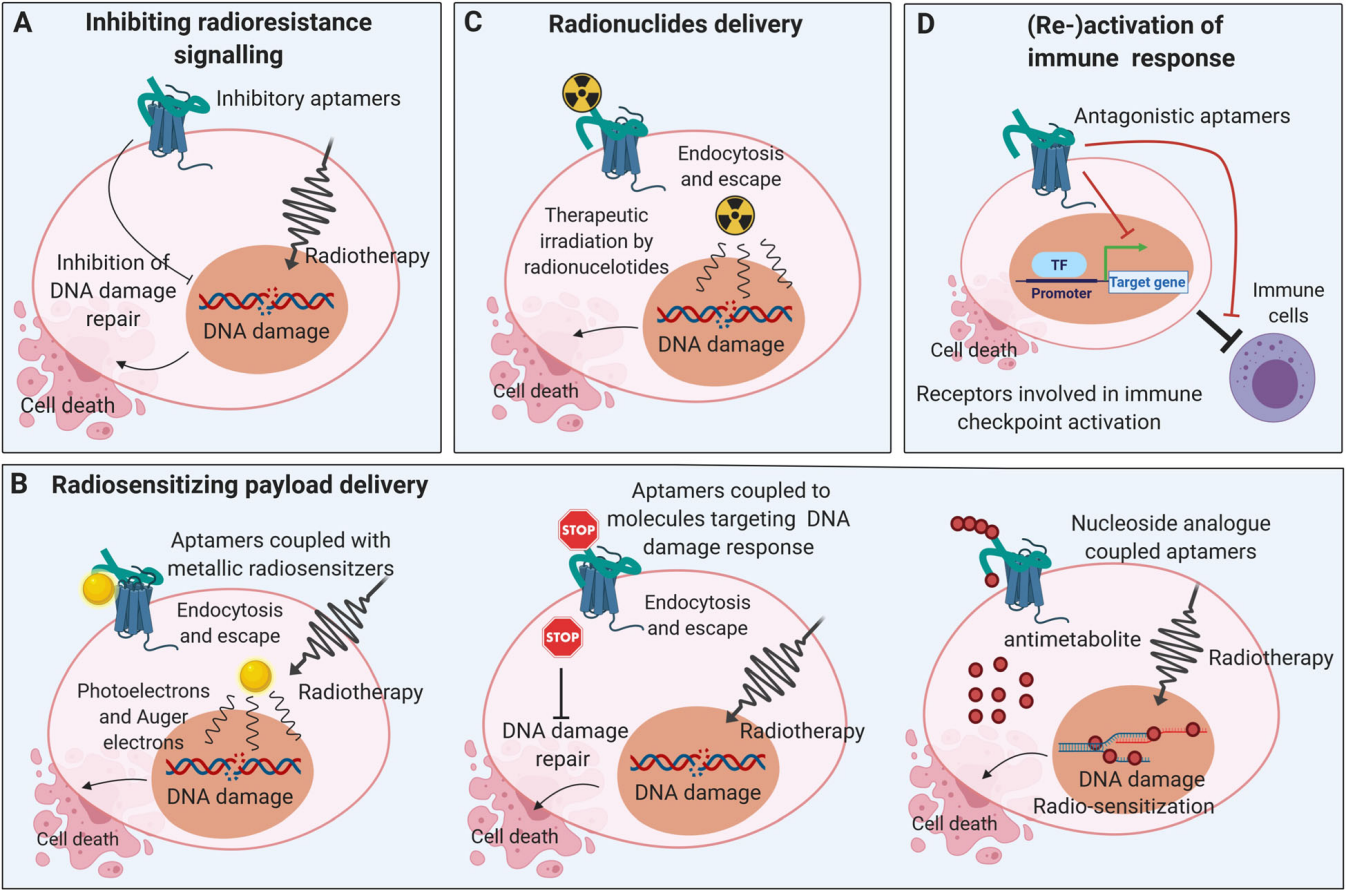
Different strategies of aptamer-based combined modality approach with radiotherapy. Aptamers specifically bind to the target structures (e.g. EGFR, PSMA, MUC-1, nucleolin, etc.), endocytosed and released in the cytoplasm following lysosomal degradation (**a)** Aptamer-binding to designated target interferes with radio-resistance signaling and sensitize radiotherapy. **b** Several radiosensitizers, such as metal formulations, siRNAs and nucleoside analogs can be coupled with aptamers for targeted delivery into cancer cells to sensitize radiotherapy. **c** Therapeutic radionuclides can be incorporated into aptamers for targeted radiotherapy. **d** These aptamers (re-)activate systemic anti-tumor immune responses by targeting immune checkpoint activation related receptors, enable antitumoral immunity and possible abscopal effects

Three strategies for the combination of aptamers and ionizing radiation can be found in the literature. The first strategy uses aptamers, which exhibit radiosensitizing properties per se without any further conjugation since binding to their designated targets interferes with radioresistance signaling (Fig. 2a). This was successfully shown in a glioblastoma (GBM) cell model, where the EGFRvIII-specific DNA-aptamer increased the radiosensitivity of EGFRvIII-expressing U87 cells, besides inhibition of proliferation, migration, and invasion [73]. The authors speculated that increased radiosensitivity with U2 treatment in GBM cells might occur through decreasing the ATP supply and inhibiting the signaling molecules in the common pathways induced by EGFRvIII and MET and thus inhibition of the DNA damage response.

The second radiosensitizing strategy utilizes aptamers as vehicles for radiosensitizing compounds (Fig. 2b). This group of aptamers can be subdivided according to the nature of their modification into aptamers which (i) deliver metal (nano-)formulations, (ii) incorporate molecules targeting the DNA damage response (DDR), and (iii) transport chemotherapeutics.

### Aptamers with metal (nano-)formulations

Aptamers of this group enforce the biological effects of radiation by physical dose enhancement via photoelectrons, Auger electrons, and secondary electrons that are generated from irradiated metal particles [74] (Fig. 2b **left panel**). This, in turn, leads to an increase in the levels of free radicals in tumor cells and enforced DNA damage. One example of aptamers in this group is the already mentioned AS1411 aptamer against cell surface nucleolin. A gold nanocluster conjugate of the AS1411 aptamer enhanced the efficacy of radiation therapy in breast tumor-bearing mice. Importantly, the authors reported a specific enrichment of the radiosensitizing gold nanoclusters in the breast tumors and a significant increase in the mean survival times of the animals [75]. Analogously, the combination of silver nanoparticles with polyethylene glycol (PEG)-functionalized AS1411 aptamer improved the outcome of radiation therapy in preclinical glioma models. Significantly prolonged median survival times were shown in animals undergoing the combined aptamer radiotherapy protocol vs. aptamer treatment alone [76]. Apart from metal formulations, the anti-MUC1 aptamer was conjugated to the radiosensitizer 1,10 phenanthroline for in-vitro radiosensitization of breast cancer cells, although the actual radiosensitizing potential of this complex has not yet been fully proven [77]. None of those mentioned above approaches used a preclinical PDAC model. Nevertheless, since the AS1411 aptamer has been shown to potently bind to PDAC cells in other studies, and since MUC1 is expressed in PDAC cells as well [78], metal (nano) formulations of both the AS1411- and the anti-MUC1- aptamer could potentially be used as radiosensitizers for pancreatic cancer.

### Aptamers with molecules targeting components of the DDR

Aptamers in this category enhance the biological effectiveness of radiotherapy by their conjugation to substances that interfere with the DNA damage response (e.g., siRNAs targeting crucial DDR regulators, Fig. 2b **middle panel**). This treatment strategy was successfully reported in an approach with targeted administration of DNA-PK silencing siRNA by aptamer-siRNA chimeras against prostate-specific membrane antigen (PSMA) in experimental models of prostate cancer. Upon aptamer treatment, an increase in radiation-induced cytotoxicity (based on specific DNA-PK knockdown) was achieved in vitro in a PSMA-positive prostate cancer cell line. The subsequent in-vivo evaluation with xenotransplants showed a PSMA-specific, significant delay in tumor growth upon the combination of aptamer treatment and radiotherapy with 6 Gy, which was not observed without irradiation or in non-PSMA expressing tumors, respectively [79, 80]. These data further strengthen the PSMA- and therefore tumor-specific nature of aptamer-mediated radiosensitization and underline the attractiveness of this approach in combination with local radiotherapy. Since PSMA expression has also been shown in PDAC [81], PSMA targeting aptamers in combination with radiotherapy may represent an exciting option for PDAC treatment and should be investigated in more detail.

An example of combining several of the mentioned aptamer strategies in one molecule is the AuNP-NUAP-STAT3d aptamer, which is targeted against nucleolin and is conjugated with gold nanoparticles and a STAT3 antagonizing decoy payload, again emphasizing the easy-to-modify nature of aptamers. The AuNP-NUAP-STAT3d aptamer was described to potently radiosensitize head and neck squamous cell carcinoma (HNSCC) cells by combining tumor-specific radical-amplifying gold nanoclusters and tumor-specific inhibition of STAT3 signaling [82].

### Targeted delivery of chemotherapeutics

As a third approach of aptamer-based radiosensitization, target-specific radiochemotherapy regimens that combine aptamers with chemotherapeutic agents are currently being investigated (Fig. 2b **right panel**). As already described, pyrimidine analogs, such as 5-FU and gemcitabine, can directly be integrated into the molecular structure of aptamers thereby optimizing therapeutic drug delivery. Analogously, aptamers were also modified for the delivery of therapeutic radionuclides (Fig. 2c). However, the latter approach is currently explored mainly for diagnostic, but not for therapeutic purposes [83]. The only therapeutic approach that has been reported so far is the combination of PEGylated liposomes loaded with the α-particle emitter 225Ac and with anti-PSMA aptamer A10 for the experimental treatment of prostate cancer models [84]. Apart from anti-PSMA aptamer A10 labeled α-particle emitter 225Ac loaded PEGylated liposomes, anti-PSMA antibody labeled ones were also generated for evaluation of their selectivity, internalization potential and killing efficacy. The results showed that the antibody was more efficient than the aptamer in terms of cytotoxicity and lethal dose values [84]. However, the comparison between antibodies and aptamers does not consider the distinction between affinity and avidity. Moreover, this difference could be explained by the double number of antibodies on the surface of the liposome compared to the number of aptamers. Each antibody has two binding sites, whereas one aptamer only has one binding site. Based on the calculation from de Almeida and colleagues for this case, antibody labeled liposomes owned 4 times higher binding capacity, but showed 1.5–1.7 times lower lethal dose values compared to aptamer labeled liposomes [83]. Thus, aptamer could be equal or better than the antibody as a delivery agent.

The third strategy is a very complex approach which made use of aptamers that were modified to (re-)activate anti-tumor immune responses (Fig. 2d). In a breast cancer mouse model, irradiation-induced upregulation of VEGF was instrumentalized to enable tumor-specific enrichment of a dual-modified aptamer against VEGF and costimulatory 4-1BB T cell antigen. The aptamer showed similar efficacy with regards to tumor control as compared to conventional, stimulatory 4-1BB antibodies, but – due to its tumor-specific delivery – relevantly less toxicity. Notably, systemic immune responses were (re-)activated, resulting in the control of distant metastatic tumor lesions outside the irradiation field. These findings underline the potential of tumor stroma-targeted aptamers to modulate anti-tumor immune mechanisms in combination with radiation [85, 86].

Although aptamers in combination with radiation therapy are rarely explored in pancreatic cancer, the strategies we summarized above provide us promising approaches to sensitize radiotherapy in several cancers. Considering the high degree of radioresistance in PDAC, these aptamer-based radiosensitizing treatments supply us with novel ideas to decrease its radioresistance and maybe improve the prognosis in the future, especially regarding the emerging new technical possibilities of image-guided irradiation (e.g. MR-Linac).

## Conclusion and perspectives

As described in this review, aptamers are very useful tools and have several applications in the diagnosis and therapy of pancreatic cancer. Unique features including ease of synthesis, high sensitivity and specificity and stability in different conditions, render aptasensors an interesting choice for the detection of biomarkers, CTCs, and CSCs. Additionally, aptamer-based biosensors represent ideal noninvasive devices for PDAC diagnosis even in early tumor stages. With help of the blind SELEX method, aptamers are able to identify novel biomarkers from cancer cells, secretomes, membranes or tissues, which can stimulate novel ideas for screening attempts and new hypotheses for mechanistic studies. Aptamer-based in-vivo imaging is an excellent way to obtain comprehensive images and molecular information without invasive biopsies. Data from aptamer-enhanced technologies will support clinical decision making and enhance the quality of precise prognostic prediction. Importantly, as aptamers can exert several ways of action, they are attractive tools for targeted therapy of PDAC, alone or in combination with standard approaches, such as radiotherapy. Aptamers can work as inhibitors and drug carriers. Their excellent performance in targeted inhibition renders them competitive alternatives to antibodies. Diverse aptamer-based drug delivery systems with incorporated nucleoside analogs, oligonucleotides, and other drugs allow aptamers to guide targeted chemotherapy or RNA interference into PDAC cells.

However, the application of aptamers has some limitations. Significant problems include degradation in blood and high renal excretion [87], although the molecules can be modified to increase plasma half-life. For RNA-aptamers, which are usually degraded by nucleases in biological media, modifications of 3′- and 5′-ends provide resistance to exonucleases, and modifications on 2′-position protect against endonucleases [88]. In order to prolong aptamer circulation in the bloodstream, conjugation with polyethylene glycol (PEG) is a commonly used strategy that increases the half-life of PEG-conjugated aptamers up to several days [89].

With the development and advancement of aptamers, these molecules are becoming an attractive platform for translational applications. To make the best use of aptamers, future directions should focus on aptamer-based biosensors and aptamer-based, targeted drug-delivery systems – particularly systems for nucleoside analogue and oligonucleotide delivery for combined modality treatment approaches.

## Data Availability

Not applicable.

## Abbreviations

5FdU: 5-Fluoro-2′-deoxyuridine
5FdUTP: 5-Fluorouracil triphosphate
5-FU: 5-Fluorouracil
AgNCs: Silver nanoclusters
ALISA: Aptamer-linked immobilized sorbent assay
ALPPL-2: Alkaline phosphatase placental-like 2
AMD: Age-related macular degeneration
anti-miRs: miRNA inhibitors
ApDCs: Aptamer-drug conjugates
ASOs: Antisense oligonucleotides
AuNPs: Gold nanoparticles
AuNS: Gold nanostars
C/EBPa-saRNA: CCAAT/enhancer binding protein-a
C4BPA: C4b-binding protein α -chain
CA 19–9: Carbohydrate antigen 19–9
CCKBR: G-protein-coupled cholecystokinin B receptor
CdS–GR: Cadmium sulfide-graphene
CEA: Carcinoembryonic antigen
CEMIP: Cell migration-inducing hyaluronan binding protein
CL: Chemiluminescence
CSCs: Cancer stem cells
CTCs: Circulating tumor cells
CTP: Cytidine triphosphate
CXCL12: C-X-C chemokine ligand 12
DDR: DNA-damage response
dFdCTP: Gemcitabine triphosphate
ECL: Electro chemiluminescence
EGFR: Epidermal growth factor receptor
ELISA: Enzyme-linked immunosorbent assay
EMT: Epithelial-mesenchymal transition
EVs: Extracellular vehicles
FDA: Food and drug administration
FOLFIRINOX: Folic acid, 5-fluorouracil, irinotecan, and oxaliplatin
GBM: Glioblastoma
GFET: Graphene field effect transistor
HNSCC: Head and neck squamous cell carcinoma
Hsp70: Heat shock protein 70
IGFBP: Insulin-like growth factor-binding protein
IL: Interleukin
LDR: Linear dynamic range
LOD: Limit of detection
MIC-1: Macrophage inhibitory cytokine-1
miRNAs: MicroRNAs
MMAE: Monomethyl auristatin E
MMAF: Monomethyl auristatin F
MMP: Matrix metalloproteinase
MRI: Magnetic resonance imaging
MSS: Microsatellite-stable
PAUF: Pancreatic adenocarcinoma up-regulated factor
PCR: Polymerase chain reaction
PD-1: Programmed death 1
PDAC: Pancreatic ductal adenocarcinoma
PD-L1: Programmed death ligand 1
PEG: Polyethylene glycol
PSMA: Prostate-specific membrane antigen
SELEX: Systematic evolution of ligands by exponential enrichment
SERS: Surface-enhanced raman spectroscopy
siRNAs: Short interfering RNAs
SPR: Surface plasmon resonance
STAT3d: STAT3 antagonizing decoy oligonucleotide
TP: Triptolide
UTP: Uridine triphosphate
VEGF: Vascular endothelial growth factor
